# Supplementary material for: Characterization of Four New Compounds from Protea cynaroides Leaves and Their Tyrosinase Inhibitory Potential
Source: Plants (Basel). 2022 Jun 30;11(13):1751. doi: 10.3390/plants11131751 (PMC9269349; doi:10.3390/plants11131751)
Supplement: Supplementary file 1 [file plants-11-01751-s001.zip › plants-1772761-supplementary.pdf]

Supplementary material

Characterization of Four New Compounds from *Protea cynaroides* Leaves and Their Tyrosinase Inhibitory Potential.

Masande Yalo [1], Masixole Makhaba [1], Ahmed Hussein [2], Rajan Sharma [2], Mkhuseli Koki [1], Ndikho Nako [1] Wilfred Mabusela [1,*].

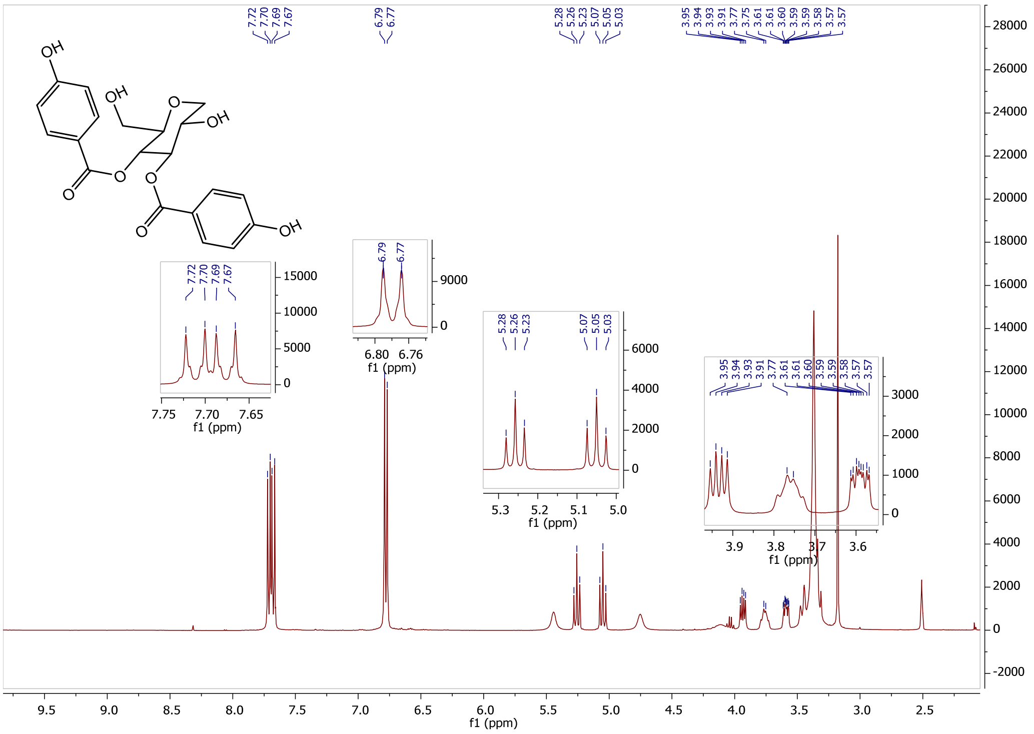

Figure S1: ^{1}H NMR spectrum proton of compound **1** in DMSO-d_6

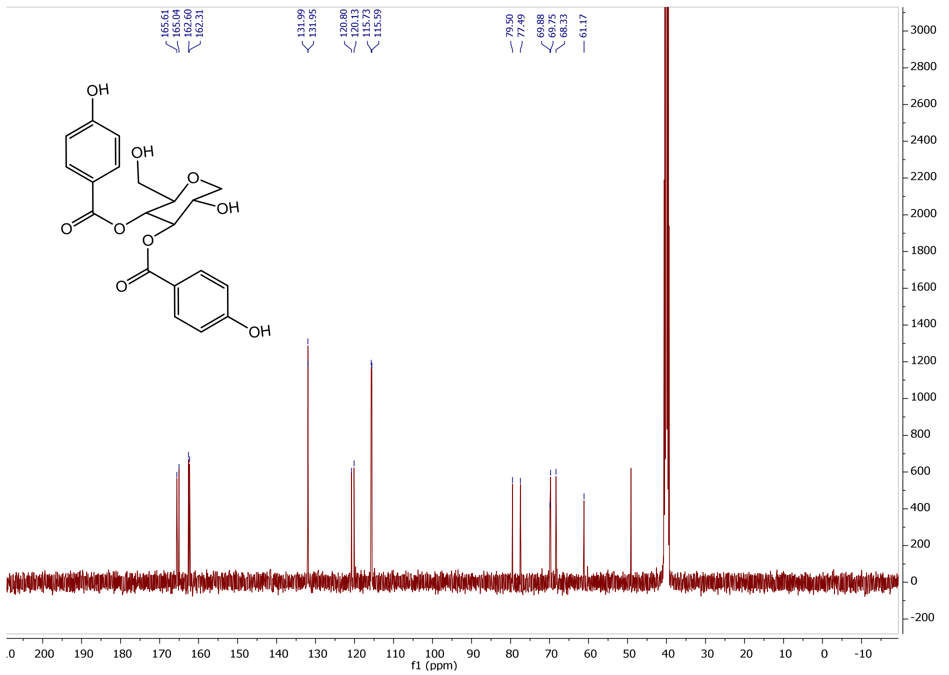

Figure S2: ^{13}C NMR spectrum of compound **1** in DMSO-d_6

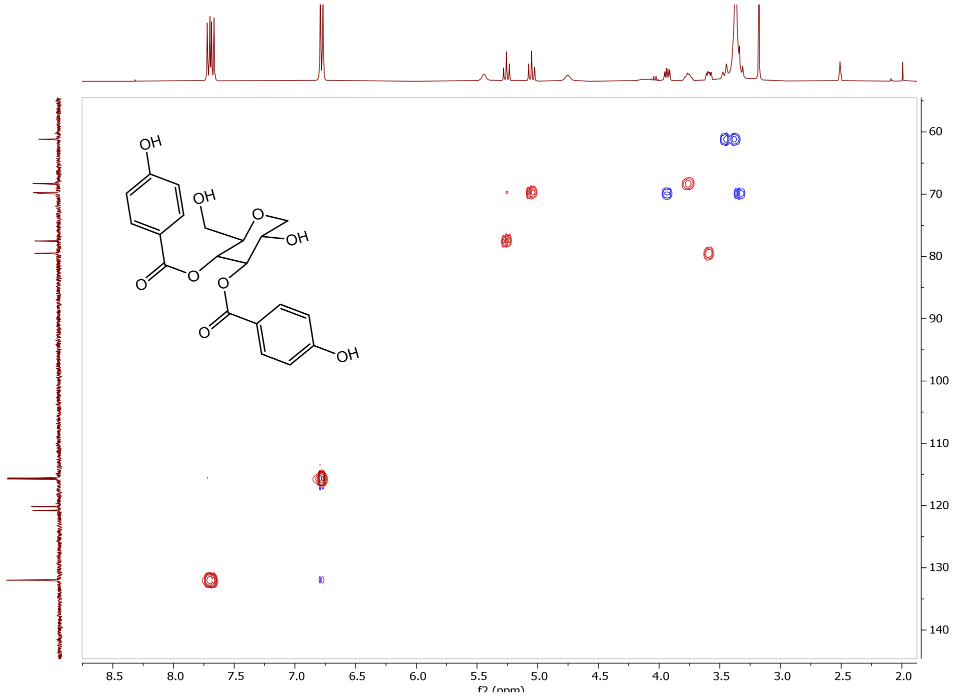

Figure S3: HSQC spectra of compound **1**

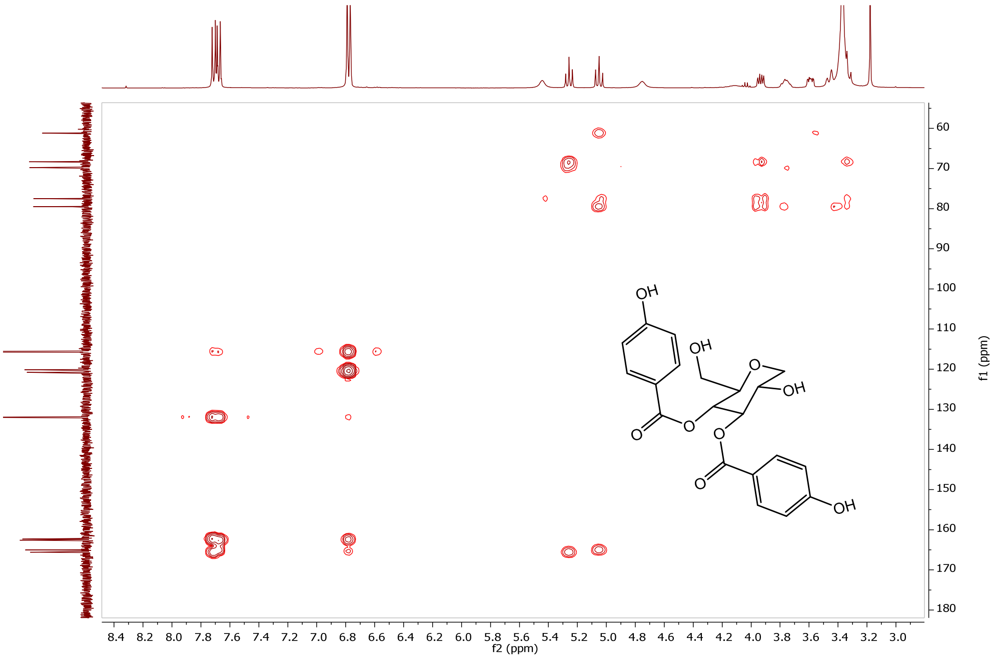

Figure S4: HMBC spectrum of compound **1**

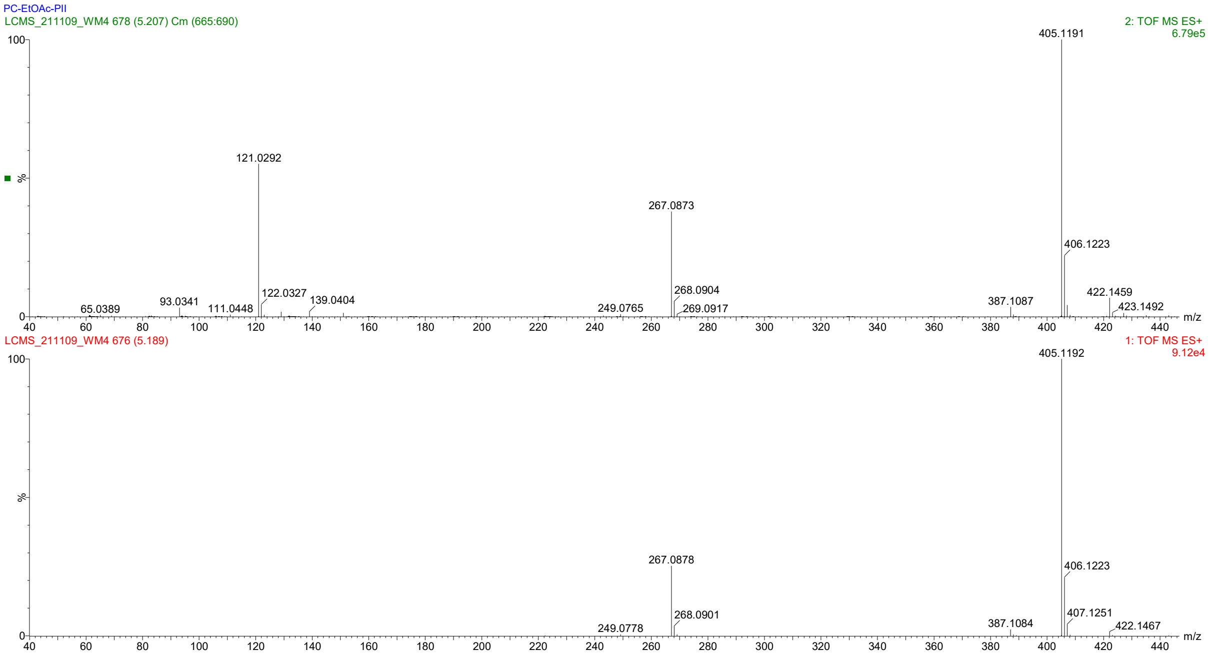

Figure S5: HRMS spectrum of compound **1**

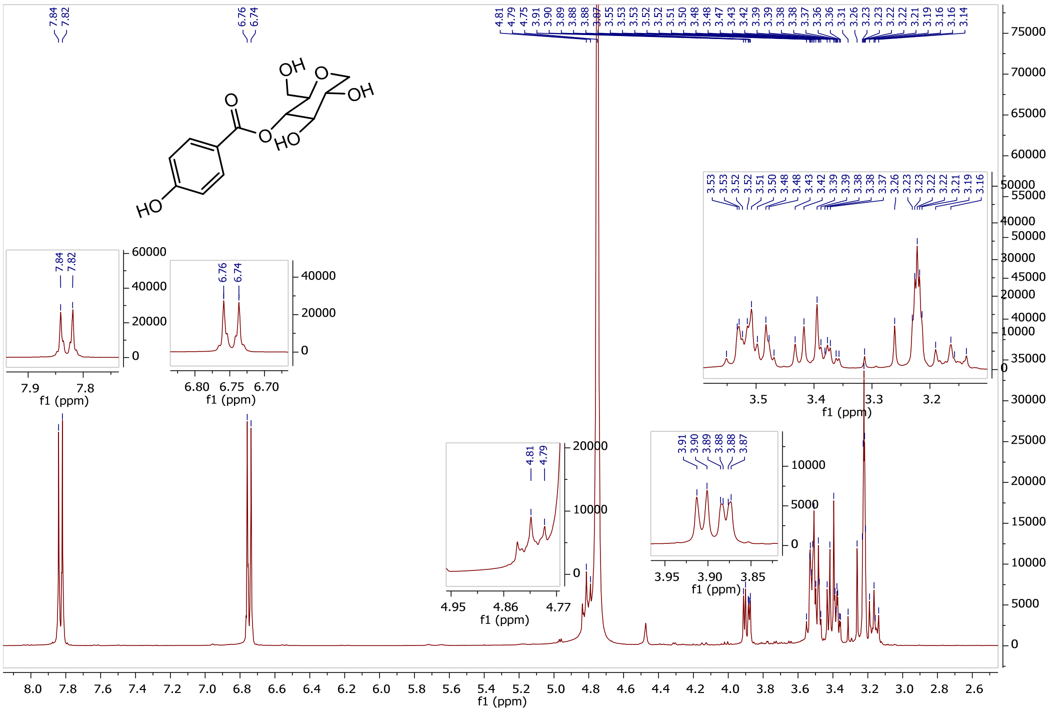

Figure S6: ^{1}H NMR spectrum proton of compound **2** in CD_3OD

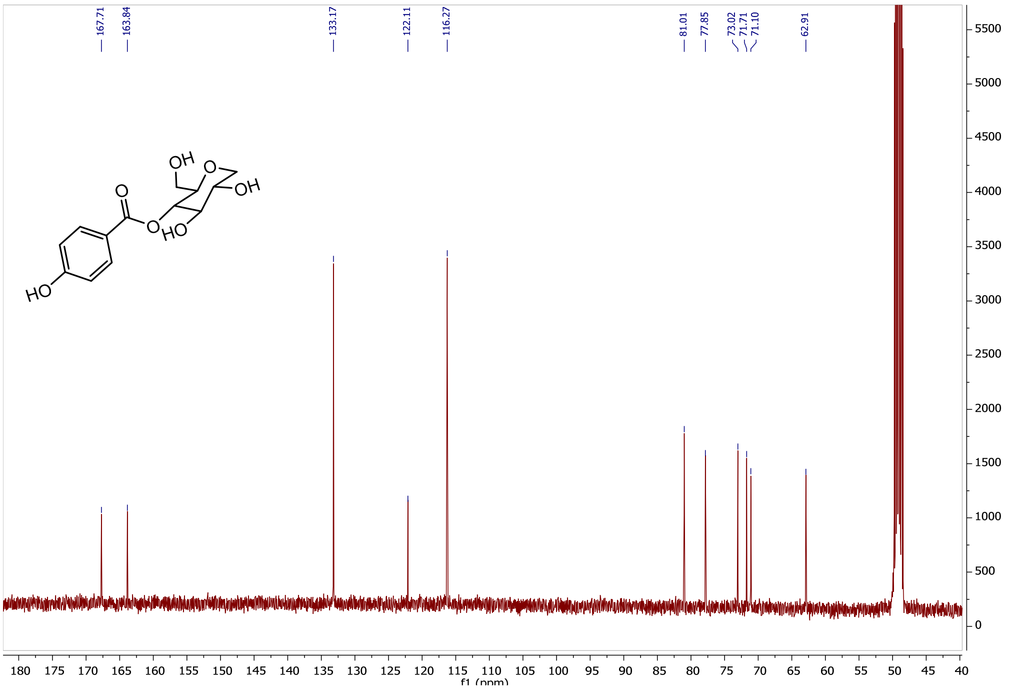

Figure S7: ^{13}C NMR spectrum of compound **2** in CD_3OD

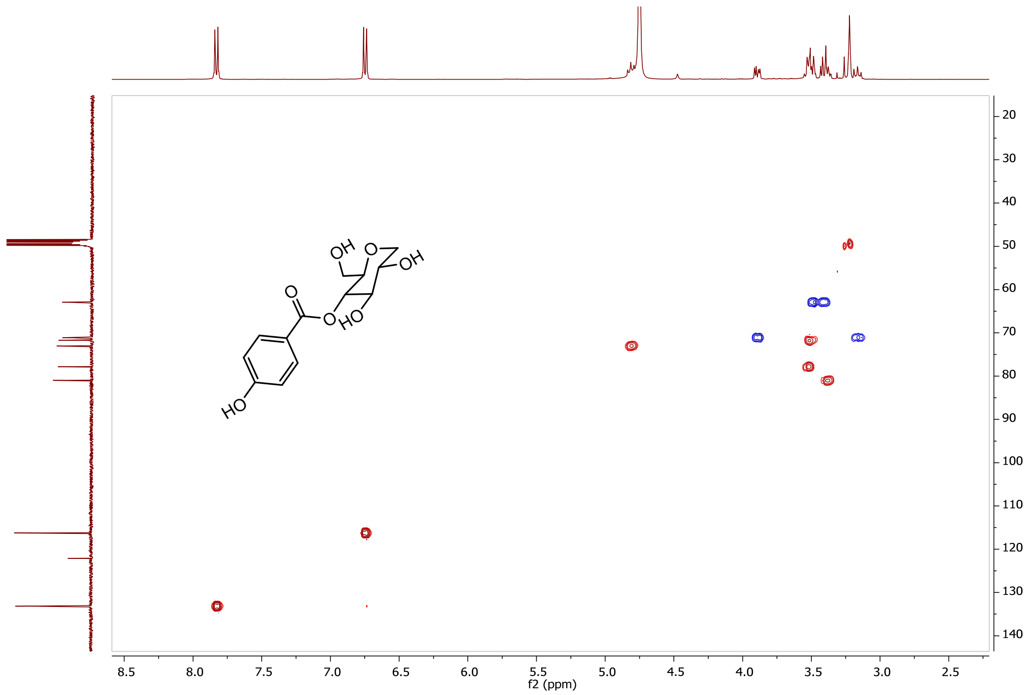

Figure S8: HSQC spectra of compound **2**

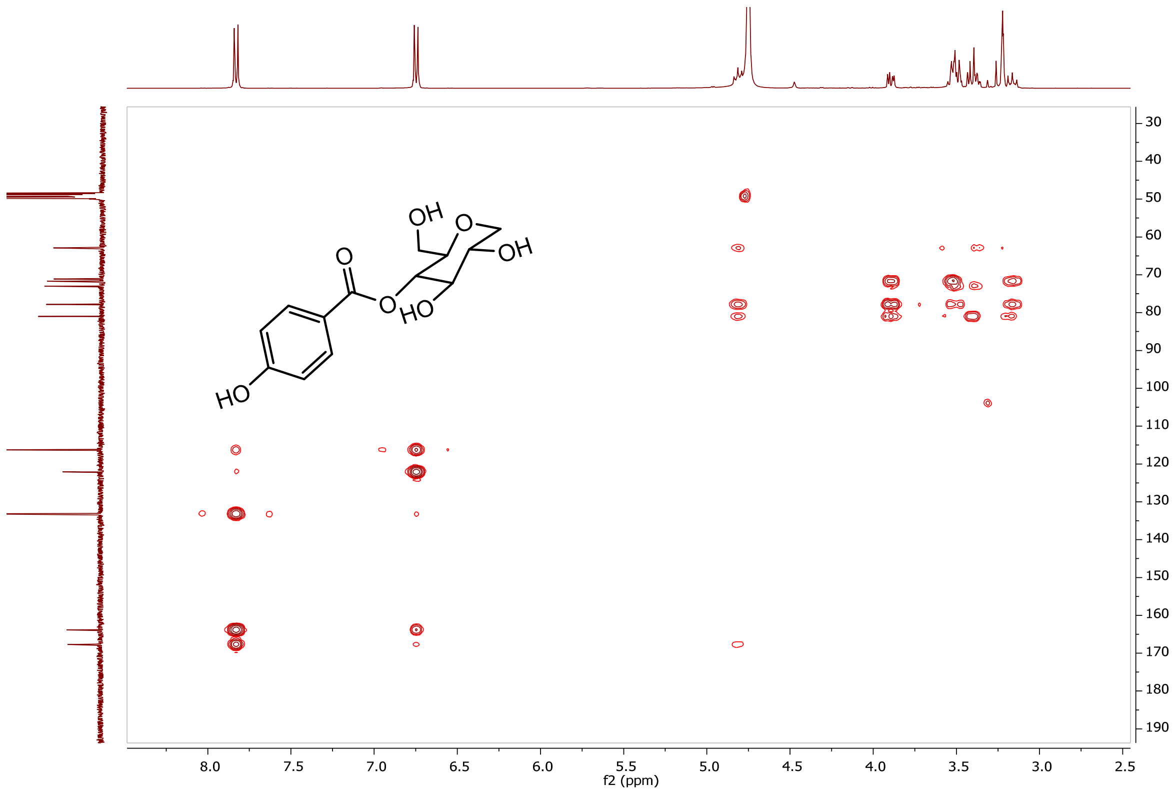

Figure S9: HMBC spectra of compound **2**

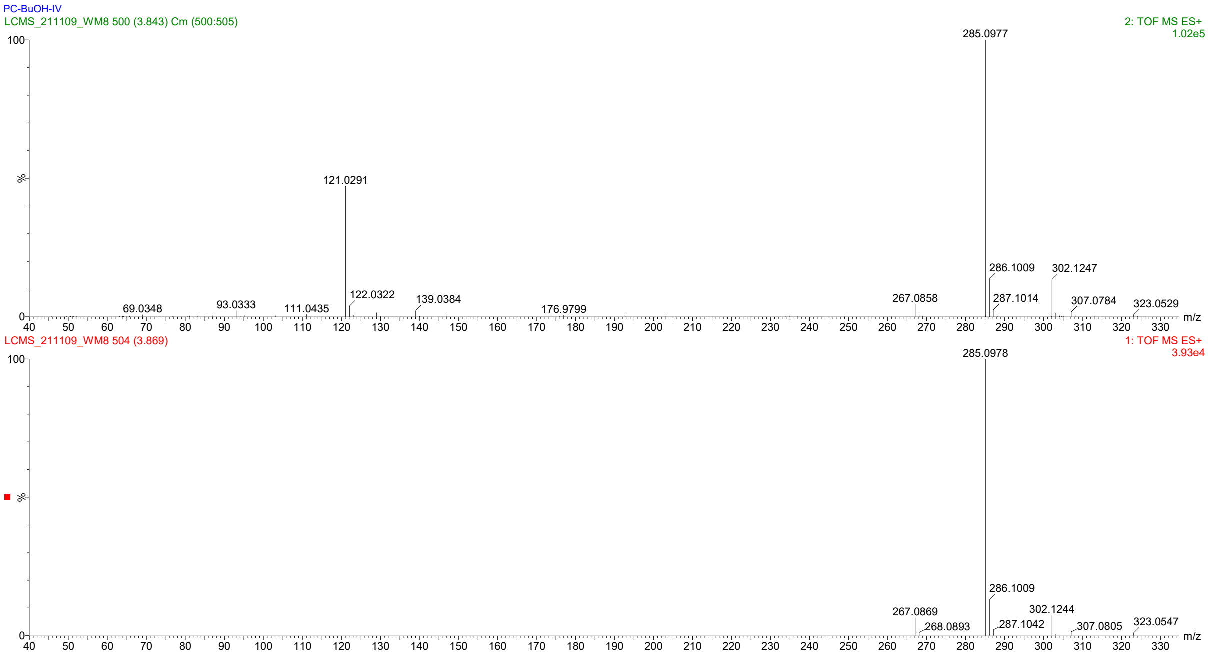

Figure S10: HRMS spectrum of compound **2**

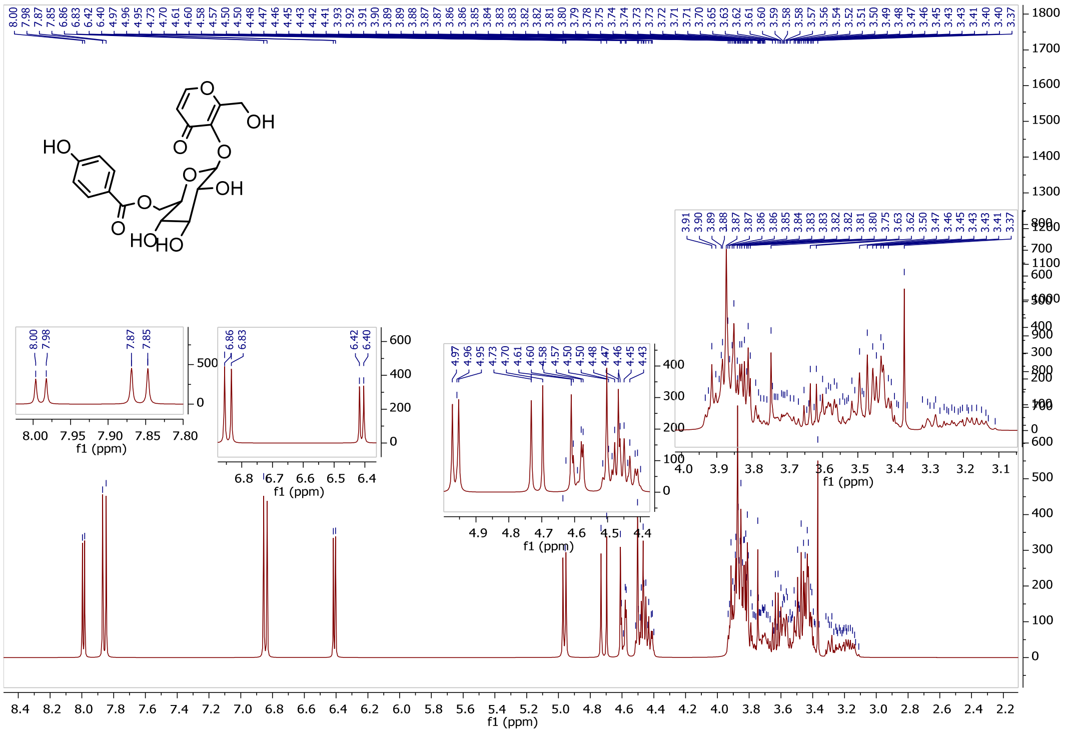

Figure S11: ^{1}H NMR spectrum of compound **3** in CD_3OD

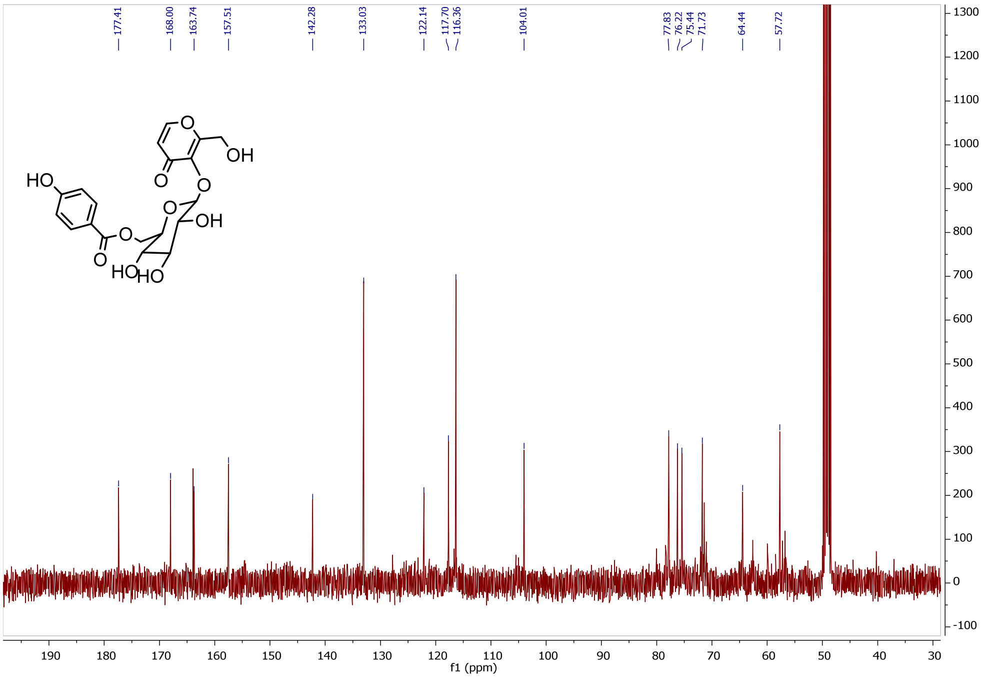

Figure S12: ^{13}C NMR spectrum of compound **3** in CD_3OD

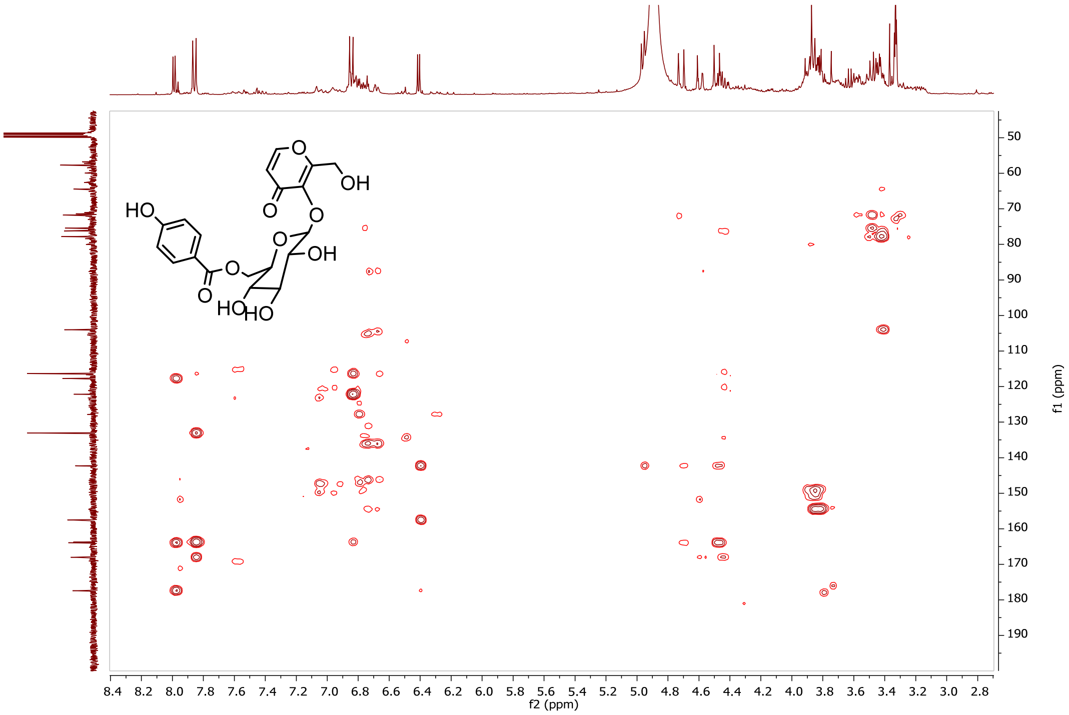

Figure S13: HMBC spectra of compound **3**

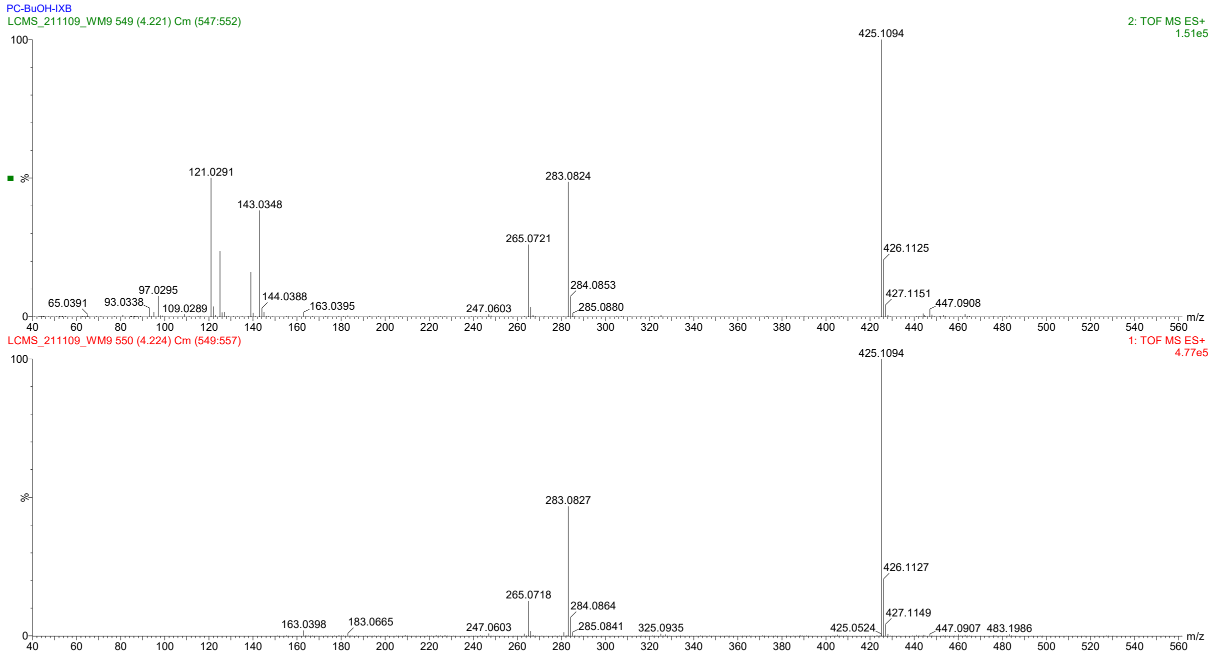

Figure S14: HRMS spectra of compound **3**

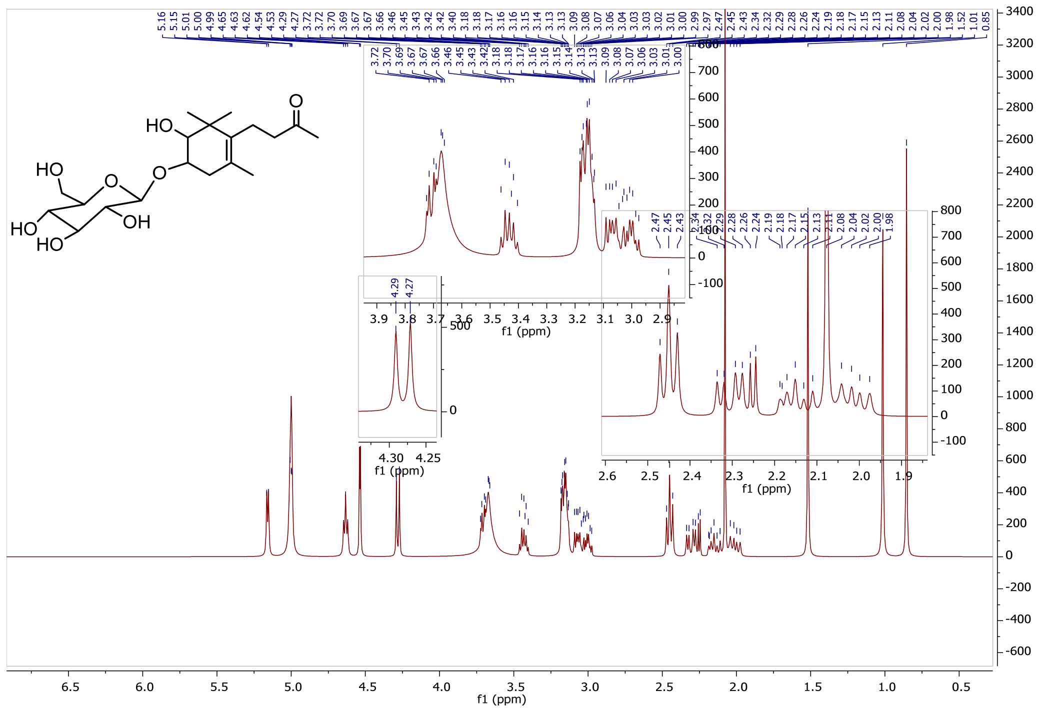

Figure S15: ^{1}H NMR spectrum of compound **4** in DMSO-d_6

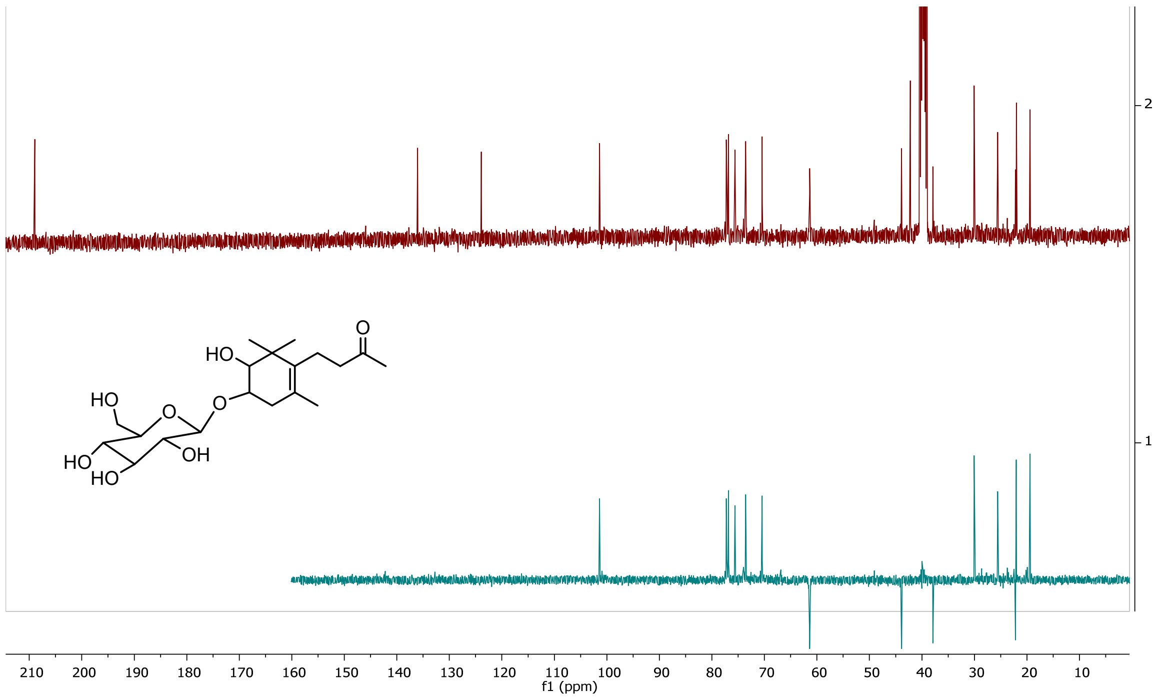

Figure 16: ^{13}C and DEPT-135 NMR spectra of compound **4** in DMSO-d_6

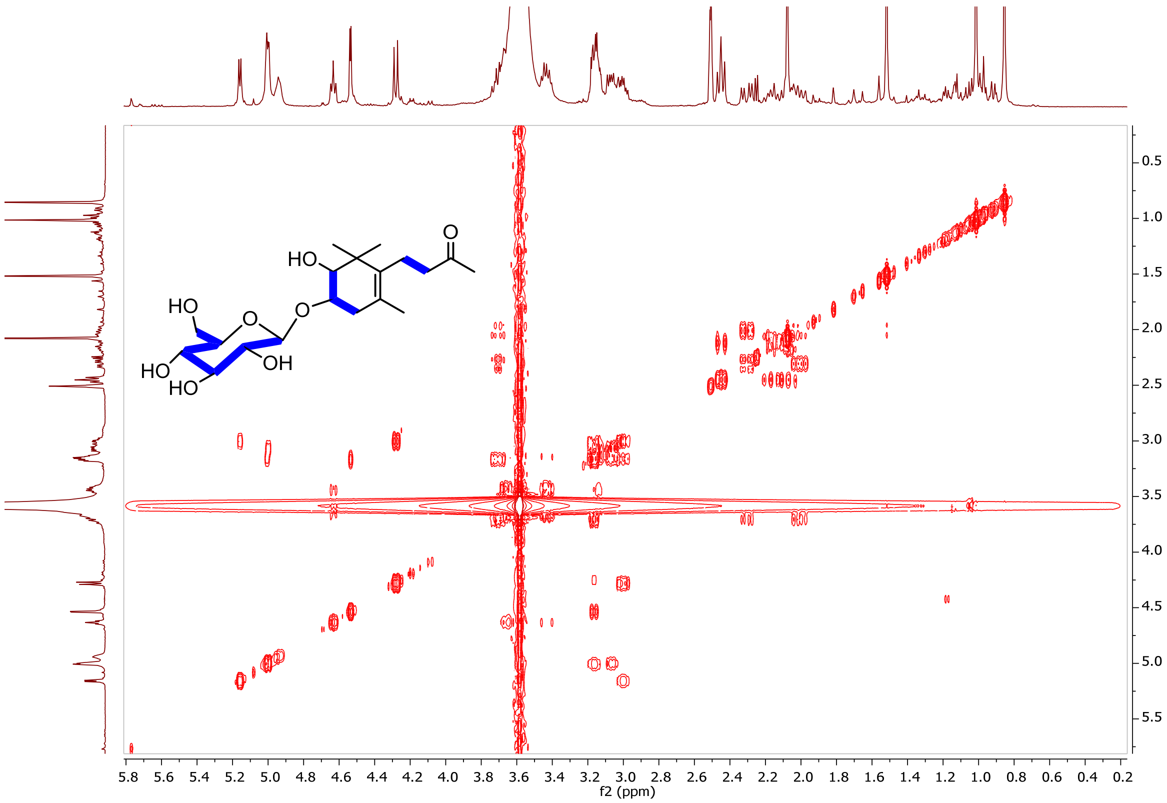

Figure S17: ^{1}H-^{1}H COSY NMR spectra of compound **4**

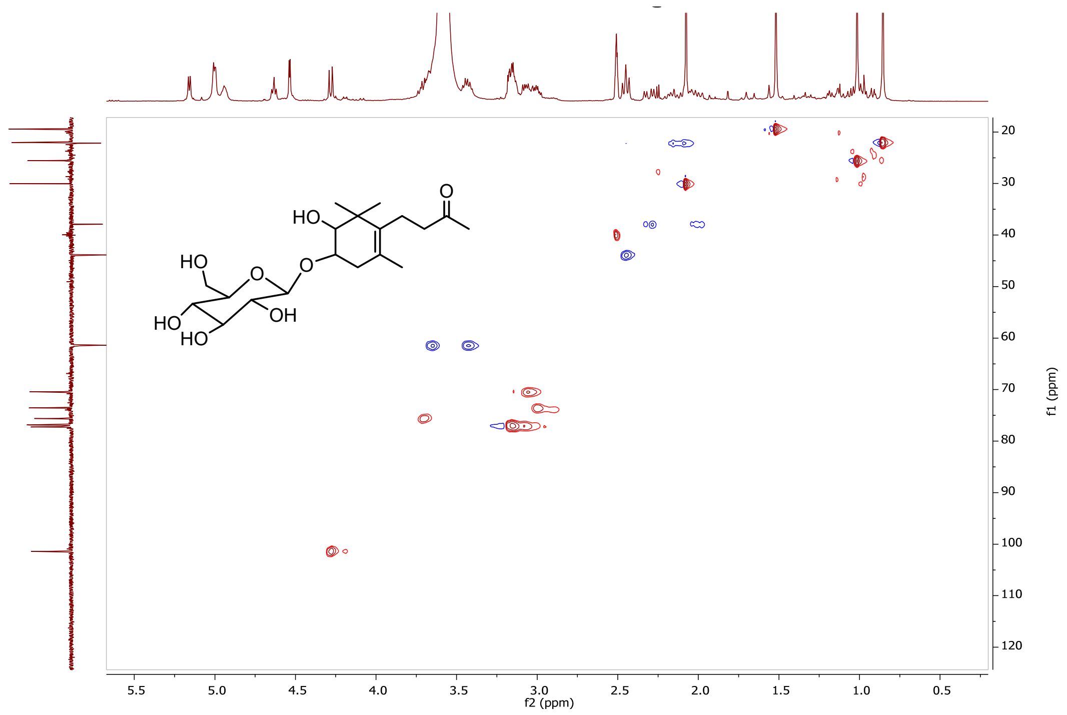

Figure S18: HSQC NMR spectra of compound **4**

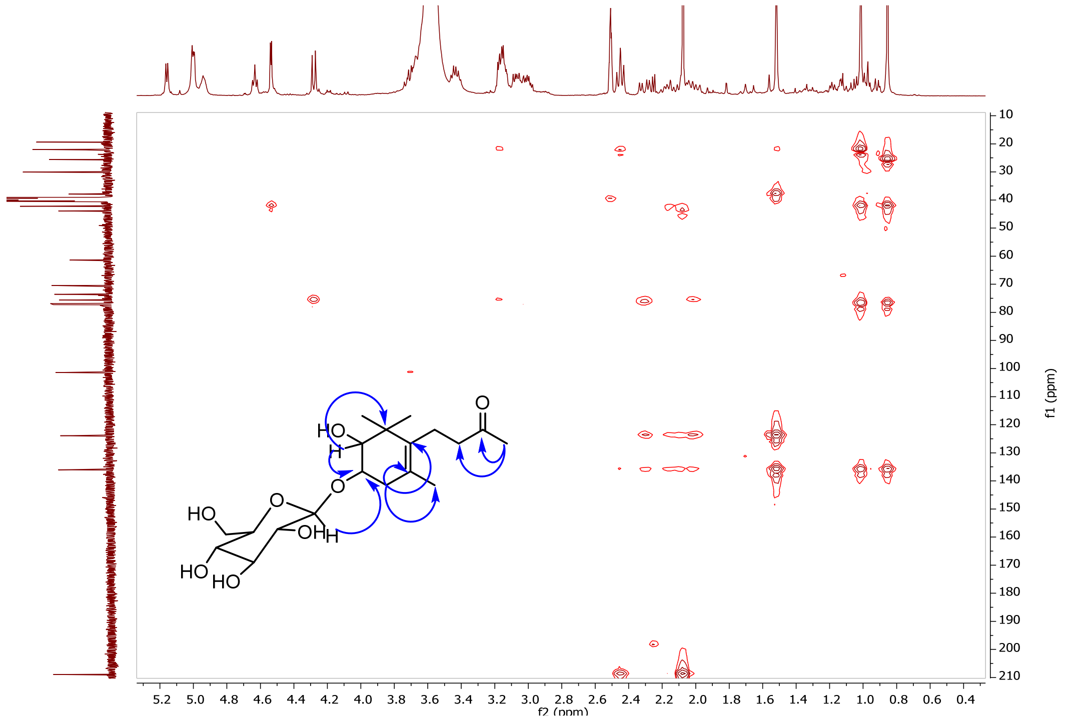

Figure S19: HMBC NMR spectra of compound **4**

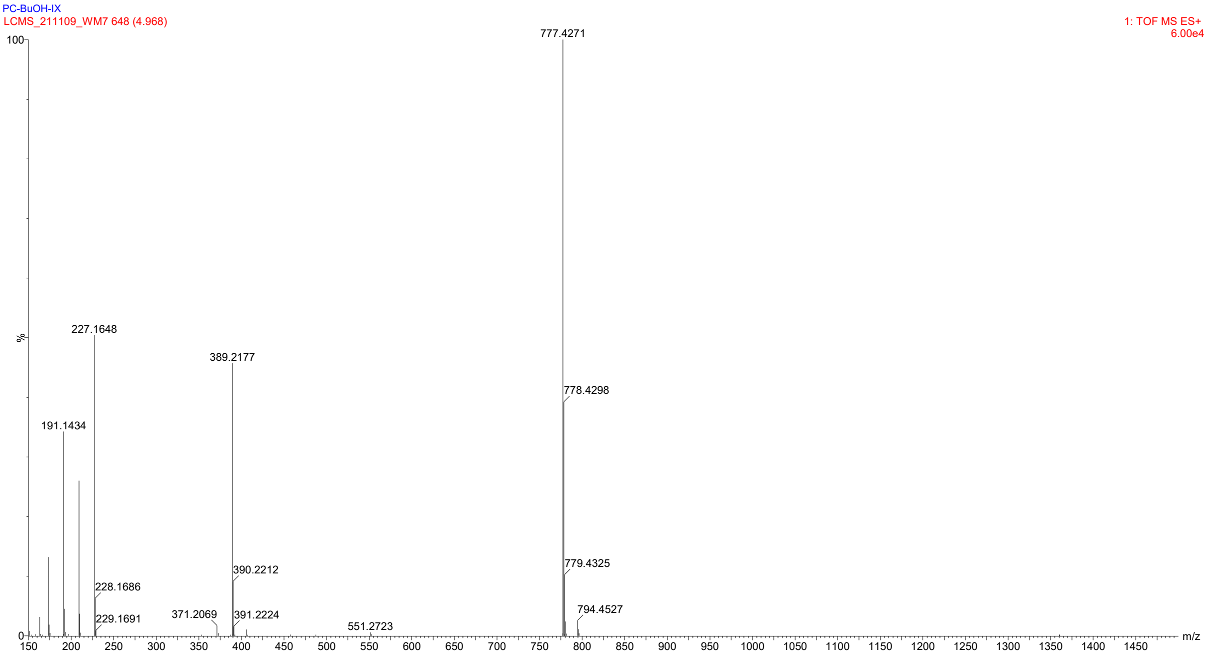

Figure S20: HRMS of compound **4**

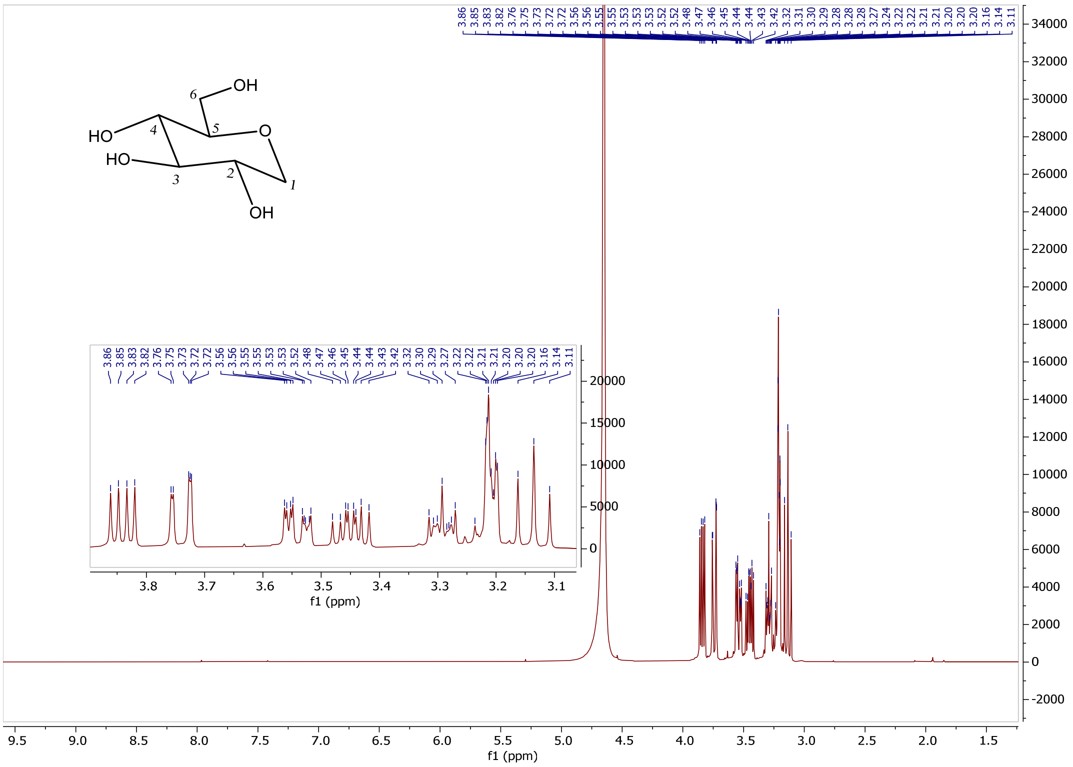

Figure S21: ^{1}H NMR spectrum proton of compound **5** in D_2O

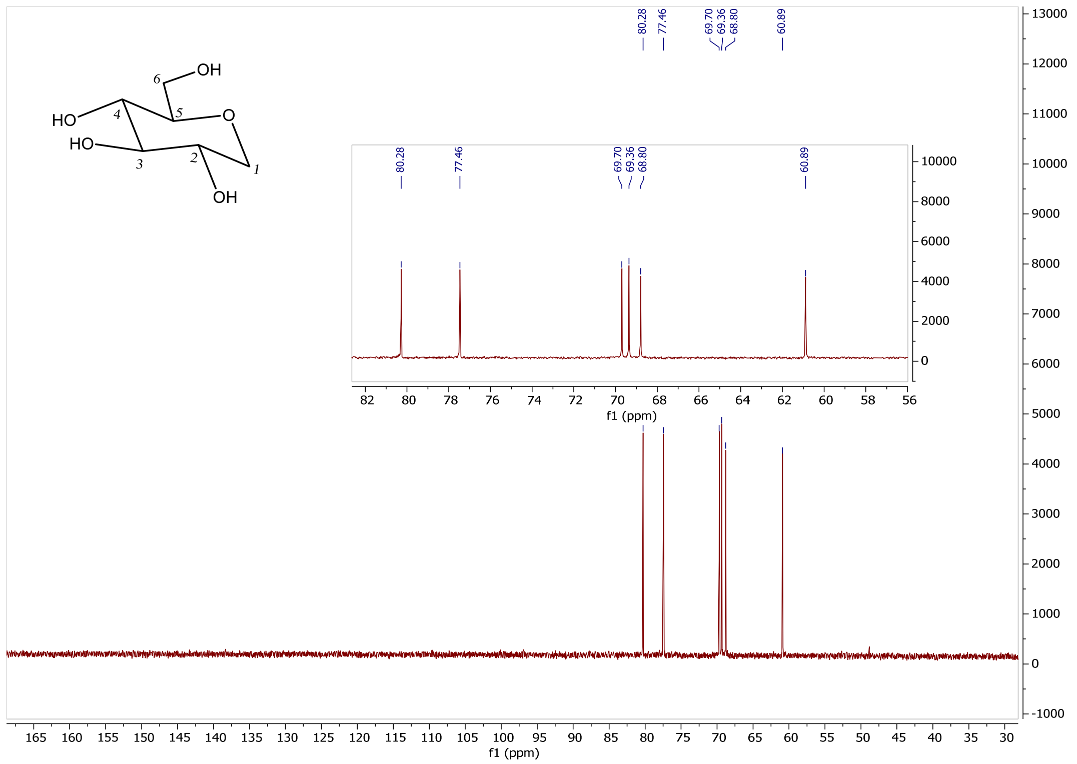

Figure S22: ^{13}C NMR spectrum of compound **8** in D_2O

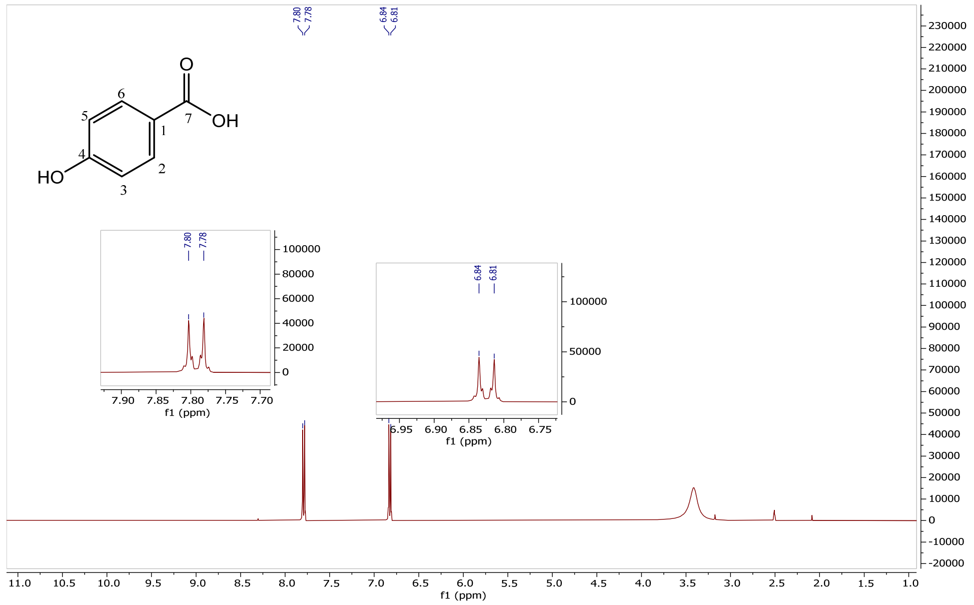

Figure S23: ^{1}H NMR spectrum proton of compound **6** in DMSO-d_6

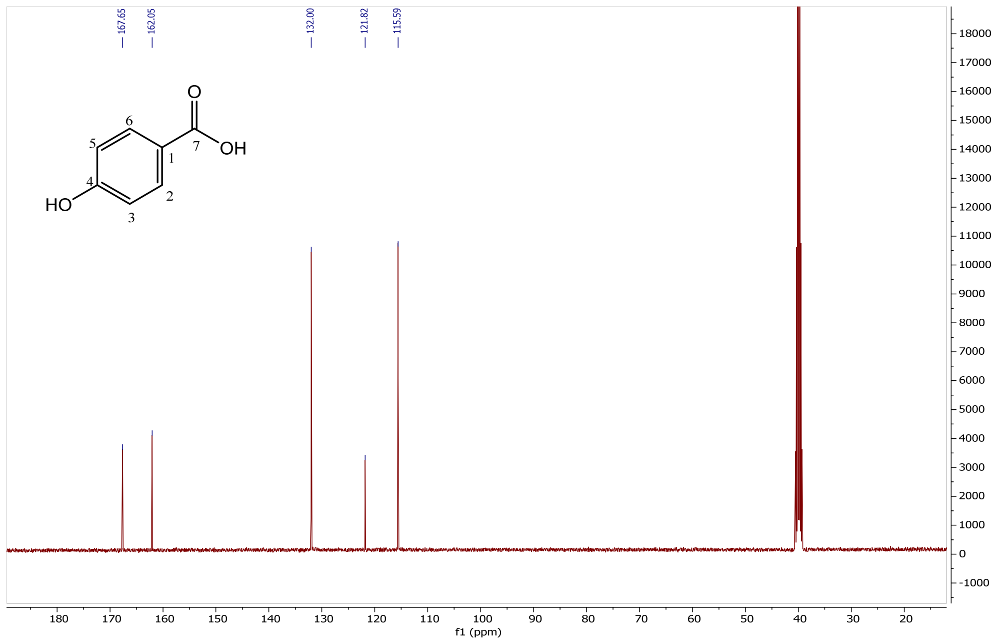

Figure S24: ^{13}C NMR spectrum of compound **6** in DMSO-d_6

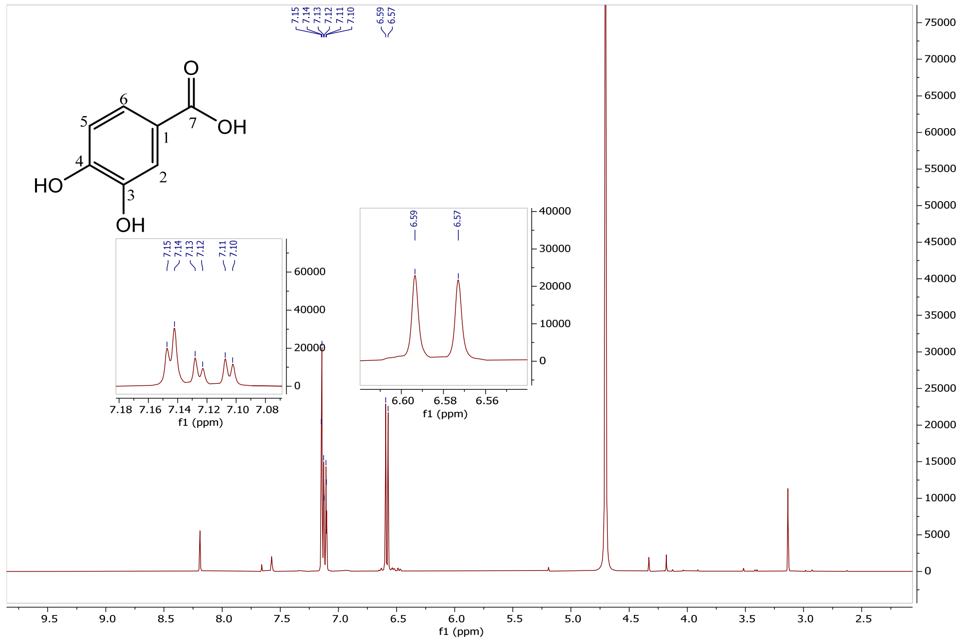

Figure S25: ^{1}H NMR spectrum proton of compound **7** in Pyridine-d_5

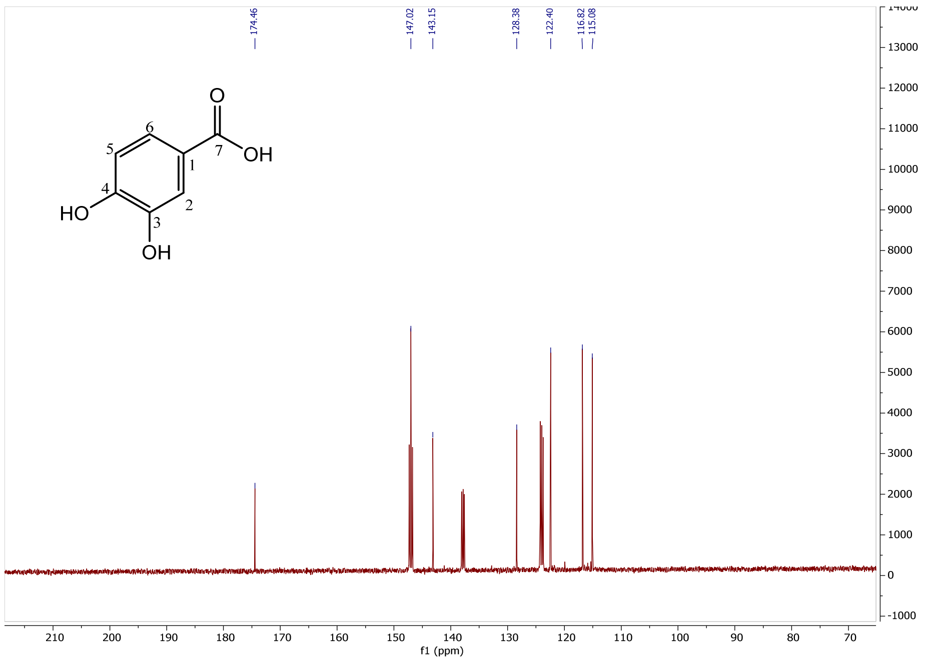

Figure S26: ^{13}C NMR spectrum of compound **7** in Pyridine-d_5

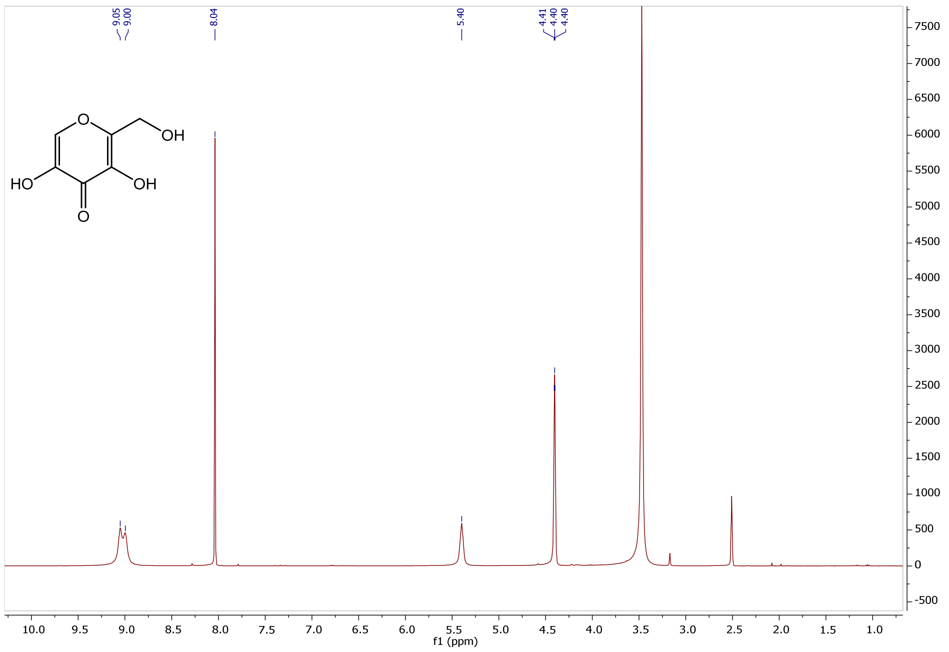

Figure S27: ^{1}H NMR spectrum proton of compound **8** in DMSO-d_6

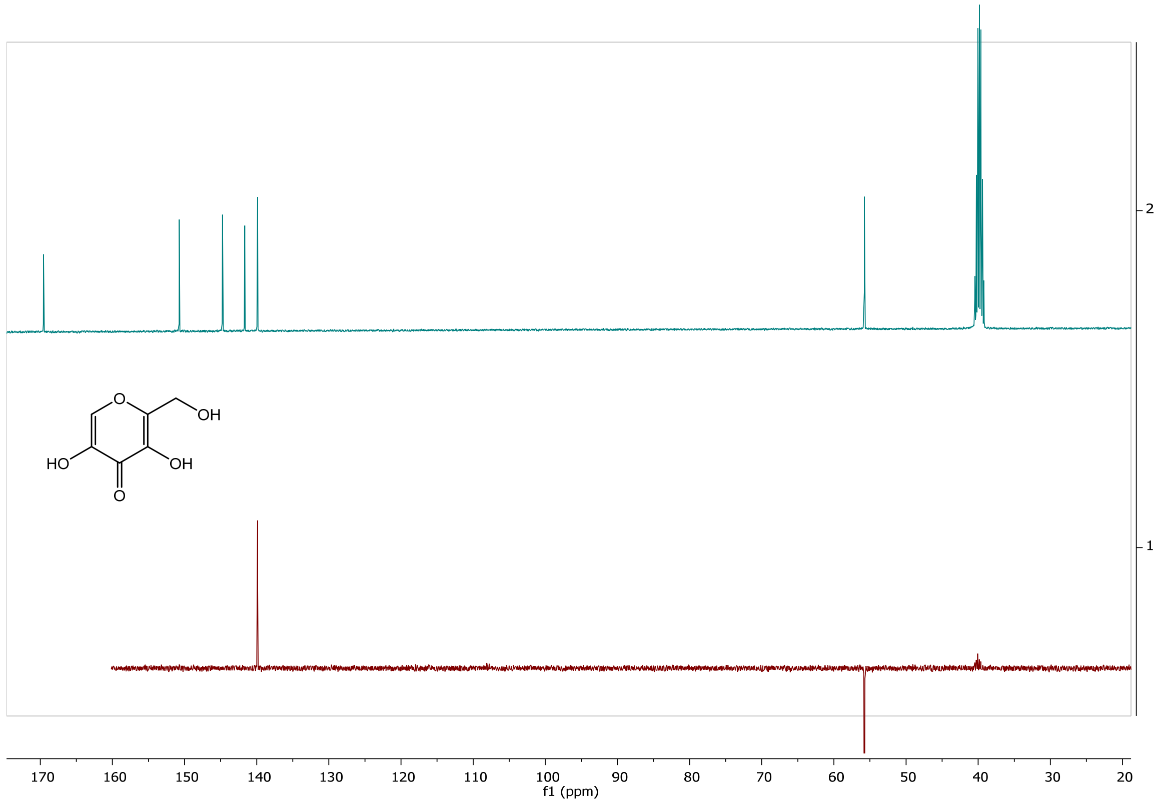

Figure S28: ^{13}C and DEPT-135 NMR spectra of compound **8**

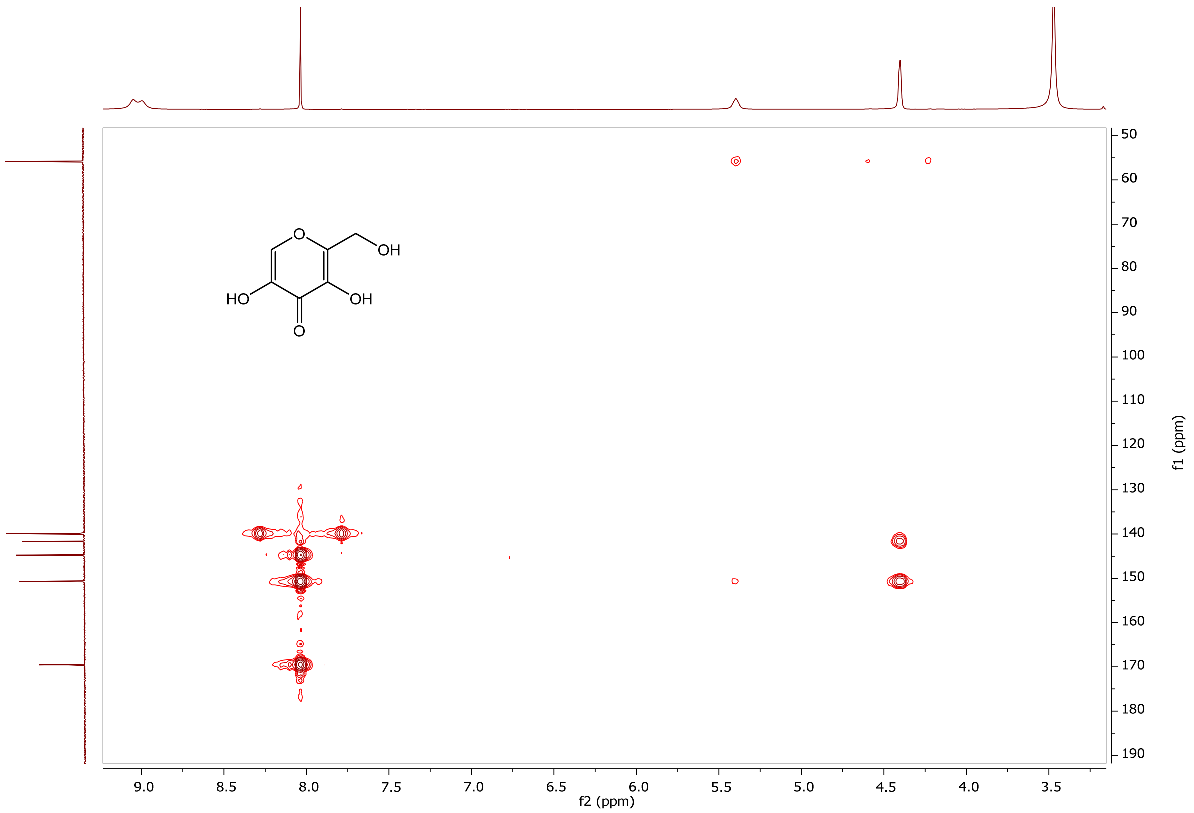

Figure S29: HMBC spectra of compound **8**
